# Biofilm Formation by Methicillin-Resistant and Methicillin-Sensitive* Staphylococcus aureus* Strains from Hospitalized Patients in Poland

**DOI:** 10.1155/2018/4657396

**Published:** 2018-12-27

**Authors:** Małgorzata Piechota, Barbara Kot, Aneta Frankowska-Maciejewska, Agata Grużewska, Agnieszka Woźniak-Kosek

**Affiliations:** ^1^Department of Microbiology, Faculty of Natural Sciences, Siedlce University of Natural Sciences and Humanities, 14 Bolesława Prusa Str., 08-110 Siedlce, Poland; ^2^Department of Agricultural Experimentation, Faculty of Natural Sciences, Siedlce University of Natural Sciences and Humanities, 14 Bolesława Prusa Str., 08-110 Siedlce, Poland; ^3^Department of Medical Diagnostics, Military Institute of Medicine in Warsaw, 128 Szaserów Str., 04-141 Warsaw, Poland

## Abstract

Biofilm-mediated infections in the hospital environment have a significant negative impact on patient health. This study aimed to investigate biofilm production in vitro and the presence of* icaABCD* genes in methicillin-resistant* S. aureus* (MRSA) and methicillin-sensitive* S. aureus* (MSSA) strains isolated from hospitalized patients. MRSA (73) and MSSA (57) strains were evaluated for biofilm production by the microtiter plate method. The presence of* ica* operon was investigated by PCR. Out of 130 strains, 99.2% were biofilm producers. Strong biofilms were formed by 39.7% of MRSA and 36.8% of MSSA strains. The highest percentage of strong biofilm producers was found among the strains isolated from sputum and tracheostomy tube (66.7%), nose and catheter (50%), throat (44.4%), and bronchoalveolar washings (43.8%). The strains isolated from bronchoalveolar washings produced significantly more biofilm than strains isolated from wound and anus. The ability of biofilm forming by fecal strains was significantly lower compared to strains from other materials. MRSA strains had significantly higher ability of biofilm formation than MSSA strains (*P* = 0.000247). The presence of* ica* operon in MRSA was detected in all strains. Comparison of strong biofilm biomass of the strains with* icaABCD*,* icaABD*, and* icaAD *revealed that strains with* icaABCD* and* icaABD* produced highly significantly more biofilm than strains with* icaAD*. Biofilm forming by both MRSA and MSSA strains indicates high ability of theses strains to persist in hospital environment which increases the risk of disease development in hospitalized patients.

## 1. Introduction

Health-care-associated infections are a live and serious problem in hospital environment. Methicillin-resistant* Staphylococcus aureus* (MRSA) is one of the major human pathogens. It is responsible for many diseases from skin infections to serious invasive infections such as pneumonia, infections of soft tissues, bones, heart valves, and even fatal septicemia in human [1]. The number of infections caused by MRSA isolates increased during the recent years and these are more frequently associated with mortality than infections caused by other bacteria.* S. aureus* is one of the most common causes of bacteremia and currently carries 20–40% mortality at 30 days despite an appropriate treatment [2]. In recent 20 years* S. aureus* infections have become more dangerous and costly to treat because of increasing prevalence of antimicrobial resistance in* S. aureus* due to the widespread use of antibiotics [3]. MRSA are resistant to *β*-lactam antibiotics but often the MRSA isolates are multidrug-resistant (MDR) because they show resistance to other antimicrobial agents, e.g., macrolides, tetracycline, aminoglycosides, chloramphenicol, and fluoroquinolones that are commonly used in therapy of infection caused by these microorganisms [4]. MRSA infections have been routinely detected in hospitalized patients including those in high-income countries. These infections are estimated to affect more than 150,000 patients annually in the European Union [5]. MRSA may infect different parts of the body including surgical wounds, skin, lower respiratory tract, and bloodstream in which they can cause different symptoms [6].

Chronic infections caused by bacterial cells forming biofilm and showing increased resistance against antimicrobial treatment are an important medical problem. It is considered that biofilms contribute to >80% of all infections in humans [7]. Colonization of medical surfaces, such as catheters and other devices, plays an important role in the problem of healthcare-associated infections because bacterial cells in biofilm show an increased resistance against classical antimicrobial treatment and host immune factors [8]. Biofilm formation by MRSA and methicillin-sensitive* S. aureus* (MSSA) strains is considered an important virulence factor influencing its persistence in both the environment and the host organism. Production of biofilm by* S. aureus* is most frequently associated with the synthesis of polysaccharide intracellular adhesin (PIA) encoded by* ica* operon [9]. Biofilm-mediated infections have an adverse effect on patient health, and therefore the main aim of this study was to investigate the capacity of clinical strains of* S. aureus* to form biofilms and the presence of* icaABCD* genes in these strains.

## 2. Materials and Methods

### 2.1. Bacterial Strains

A total of 130* S. aureus* strains from clinical materials from humans such as swabs from wound (25), nose (8), anus (16), throat (9), tracheostomy tube (3), and catheter (2) and samples of blood (13), urine (5), bronchoalveolar washings (32), purulence (6), sputum (3), and other samples (8) were used in this study. The strains were obtained from hospitals in Siedlce and Warsaw (Poland) in 2015-2017. The methods of identification of isolates as* S. aureus* were described previously [10]. The resistance of the strains to methicillin was tested with a disc diffusion method according to CLSI [11]. The* mecA* gene responsible for resistance against *β*-lactam antibiotics was identified by PCR [12].

### 2.2. Biofilm Formation Assay

Each strain was grown on Tryptic-Soy Agar (TSA; BBL, Becton Dickinson, Sparks, Md.) with 0.5% glucose at 37°C for 18 h. After that, bacterial cells were transferred to Tryptic-Soy Broth (TSB) with 0.5% glucose in order to prepare cell suspension containing about 10^8^CFU/mL. Subsequently, bacterial cell suspension (200 *μ*L) was inoculated in eight replicates to wells of a tissue culture polystyrene 96-well plate (Nunclon, Roskilde, Denmark). Biofilms were developed for 48 h at 37°C. After this time, the medium was removed and nonadherent bacterial cells were discarded by washing the biofilms twice with 250 *μ*L of sterile phosphate buffered saline (PBS, pH 7.4). Biofilm was fixed with 200 *μ*L of methanol per well for 15 min and stained for 5 min with 200 *μ*L of 1% crystal violet per well (Sigma-Aldrich, Steinheim, Germany). After rinsing with distilled water the plates were air dried. After that, colorant was solved in 96% ethanol to measure absorbance at 492 nm in microplate reader (Apollo LB913, Berthold Technologies, Germany). Each assay was performed three times and the results were averaged. Values of absorbance ≥0.12 were regarded as biofilm positive, <0.2 were considered weak producers, 0.2-0.4 were moderate producers, and >0.4 were considered strong producers [13].

### 2.3. DNA Isolation

Genomic DNA was isolated from* S. aureus* strains by using the NucleoSpin Microbial DNA (Macherey-Nagel GmbH&Co.KG, Düren, Germany) according to the manufacturer's protocol. 2.5 *μ*L of the total extracted material from each test sample was used as a template DNA for PCR application.

### 2.4. Primers and PCR Conditions

The primers specific for the* icaA*,* icaB*,* ica*C, and* icaD* synthesized by DNA-Gdańsk (Gdańsk, Poland) are listed in Table 1.

The monoplex PCR for each gene was performed in a 25-*μ*L volume containing 2.5 *μ*L of DNA template, 1×PCR buffer, 0.2 mM each dATP, dCTP, dGTP, and dTTP (Fermentas, Lithuania), the specific primers at 100 nM, and 1 U of REDTaq Genomic DNA polymerase (Sigma-Aldrich, Germany). The amplification was carried out in the following conditions: initial denaturation (94°C, 4 min), followed by 35 subsequent cycles consisting of denaturation (94°C, 30 s), primer annealing (56°C, 30 s), extension (72°C, 1 min), and final extension (72°C, 10 min).

Amplifications were carried out in the Eppendorf Mastercycler nexus gradient (Germany). The PCR products were analyzed by electrophoresis in 1.5% agarose gels stained with ethidium bromide. Molecular size markers (Sigma-Aldrich) were also run for product size verification. The gel was electrophoresed in 2 × Tris-borate buffer at 70 V for 1.5 h.

### 2.5. Statistical Analysis

Comparisons were analyzed by using Statistics 13.1 (Analytical Software, Tallahassee, FL). The comparison of average values of absorbance at 492 nm between two groups was performed by Mann-Whitney U-test. In case of nonparametric tests of three or more groups, Kruskal-Wallis test was performed. In the analysis, multiple comparisons of average ranks were used.  *χ*^2^ test was used to determine the relationships between the presence of* ica* operon genes and susceptibility or resistance to methicillin.

## 3. Results

*S. aureus* strains included in this study were collected from individuals in hospitals in Siedlce and Warsaw (Poland) between 2015 and 2017 and were divided into MSSA (57 strains) and MRSA (73 strains).* S. aureus* strains were evaluated for biofilm formation on polystyrene surface. Out of 130 strains, 99.2% were biofilm producers. Only one strain isolated from anus did not produce biofilm. Based on absorbance values at 492 nm, the strains were considered weak, moderate, and strong producers of biofilm. About 37% of strains were strong producers, while above 49% and above 13% of strains were moderate and weak producers, respectively. The highest percentage of strong biofilm producers was among the strains isolated from sputum and tracheostomy tube (66.7%), nose and catheter (50%), throat (44.4%), and bronchoalveolar washings (43.8%) (Table 2).

All strains from tracheostomy tube and catheter were MRSA. Among the strains from throat about 89% were resistant to methicillin. The percentage of strains resistant to this group of antibiotics isolated from sputum was equal to 66.7%, while among strains from nose and bronchoalveolar washings it was 62.5%.

**Figure 1:**
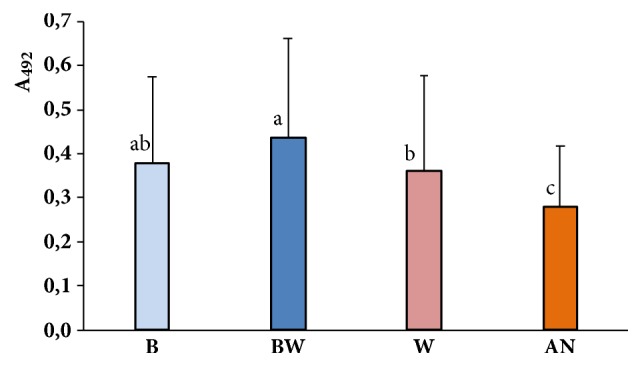
Biofilm-forming ability of the four most numerous groups of* S. aureus* strains isolated from blood (B), bronchoalveolar washings (BW), wound (W), and anus (AN). Values of absorbance at 492 nm (A_492_) were compared by using Kruskal-Wallis test. In analysis, multiple comparisons of average ranks at* P* ≤ 0.05 were used. The designation with different letters indicates that biofilm biomass formed by different groups of strains differs significantly.

The comparison of biofilm biomass (absorbance at 492 nm) of four most numerous groups of strains isolated from blood, bronchoalveolar washings, wound, and anus revealed that strains isolated from bronchoalveolar washings produced significantly more biofilm than strains isolated from wound and anus but difference between biofilm biomass of strains from bronchoalveolar washings and from blood was not statistically significant. No statistically significant difference in biofilm production was observed between strains isolated from blood and wound. While, the ability of biofilm forming by the strains isolated from anus was significantly lower comparing to strains isolated from other clinical materials (Figure 1).

**Figure 2:**
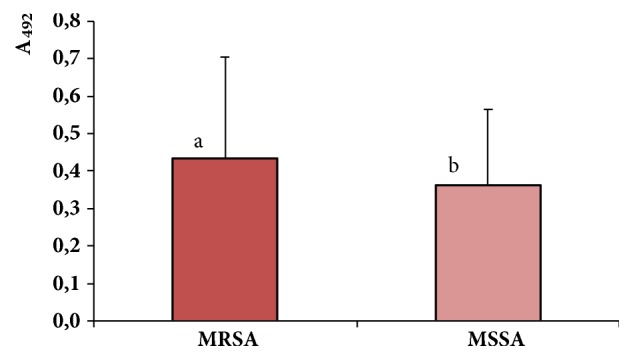
Comparison of ability of biofilm forming by MRSA and MSSA strains. Two group comparisons of average values of absorbance at 492 nm (A_492_) were analyzed by using Mann-Whitney U-test. The designation with different letters indicates that biofilm biomass formed by MRSA and MSSA strains differs significantly at* P* = 0.000247.

The majority of the 73 MRSA strains examined (47.9%) formed moderate biofilms. The strong biofilms were formed by 39.7% of the MRSA strains, while 11% of strains were weak producers of biofilm. One methicillin-resistant strain isolated from anus did not produce biofilm. A large group of the 57 MSSA strains examined (45.6%) also formed moderate biofilms, while 36.8% and 17.6% of MSSA strains were strong and weak producers, respectively. Comparison of biofilm biomass (absorbance at 492 nm) using the statistical test showed that MRSA strains had significantly higher ability of biofilm formation than MSSA strains (*P* = 0.000247) (Figure 2).

**Figure 3:**
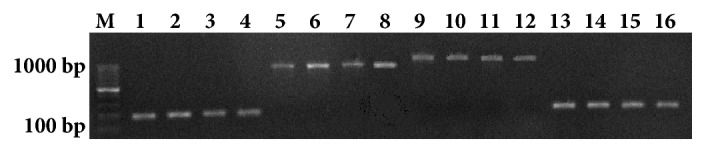
Electrophoresis in 1.5% agarose gel PCR products obtained by using specific primers for* icaABCD *genes. Lines M: molecular weight markers (1000, 900, 800, 700, 600, 500, 400, 300, 200, 100 bp; GenoPlast Biochemicals, Poland), Lines 1–4: products (188 bp) obtained by using specific primers for* icaA* gene; Lines 5–8: products (900 bp) obtained by using specific primers for* icaB* gene; Lines 9–12: products (1100 bp) obtained by using specific primers for* icaC* gene; Lines 13–16: products (198 bp) obtained by using specific primers for* icaD* gene.

The presence of the* ica* operon in the investigated strains was demonstrated by amplification of the specific fragments for* icaA* (188 bp),* icaB* (900 bp),* icaC* (1100 bp), and* icaD *(198 bp) (Figure 3).

**Figure 4:**
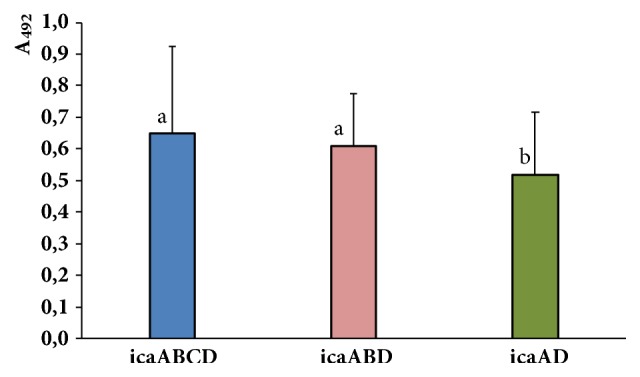
Comparison of ability of strong biofilm forming by* S. aureus* strains with* icaABCD*,* icaABD*, and* icaAD* genes. Values of absorbance at 492 nm were compared by using Kruskal-Wallis test. In analysis, multiple comparisons of average ranks at* P* ≤ 0.01 were used. The designation with different letters indicates that biofilm biomass formed by strains with different genes differs significantly.

The* icaABCD* genes were present in 67 (51.5%) strains, while the* icaABD* and* icaAD* genes were detected in 34 (26.1%) and 20 (15.4%) strains, respectively. Few strains harbored only* icaA *(3.1%) or* icaACD* (1.5%) genes. In remaining three strains the* icaAB*,* icaBD*, and* icaBCD *genes were detected. The comparison of strong biofilm biomass of the strains with* icaABCD*,* icaABD*, and* icaAD *revealed that strains with* icaABCD* and* icaABD* produced highly significantly more biofilm than strains with* icaAD* (*P* = 0.000027 and* P *= 0.003027, respectively) (Figure 4). No statistically significant difference in biofilm production was observed in strains that harbored these genes and formed moderate and weak biofilm.

**Figure 5:**
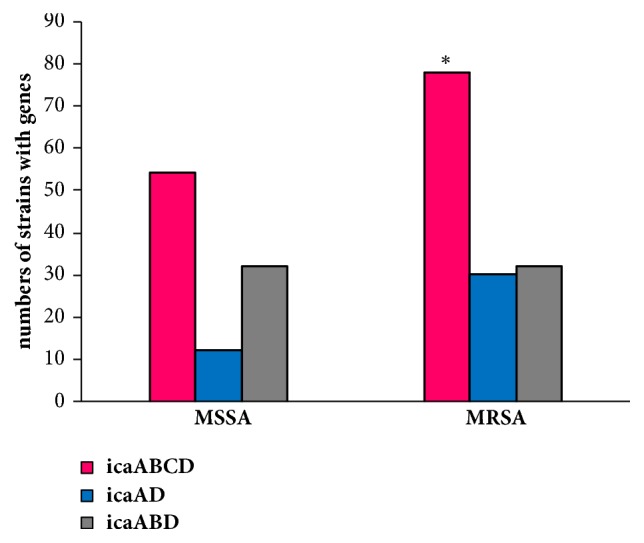
The relationships between the presence of* ica* operon genes and susceptibility or resistance to methicillin. *∗* The* icaABCD* genes were significantly associated with the MRSA strains (*P* = 0.04) (*χ*^2^ test).

The occurrence of* icaABCD* genes was significantly associated with the MRSA strains (*P* = 0.04). The* icaABCD* genes were also present in small group of MSSA strains (Figure 5).

## 4. Discussion

Biofilm production by* S. aureus* has been identified as an important factor of pathogenesis, protecting against the immune system and antibiotics, and is considered to be responsible for chronic or persistent infections [16].* S. aureus* forming biofilm causes many diseases including septicemia, endocarditis, and osteomyelitis and is a serious problem in nosocomial infections caused by* S. aureus* [17, 18]. In our study we investigated the ability to form biofilm by* S. aureus* strains isolated from clinical materials from hospitalized patients and almost all strains (99.2%) adhered to polystyrene but differed in the ability of producing biofilm, whereas Agarwal and Jain (2013) [19], who grouped* S. aureus* in three categories, showed that isolates showing biofilm producing potential occurred more often among invasive (from blood) and colonizing (from intravenous device) isolates than in group of commensal isolates (from skin or nose). Our strains also originated from diverse sources but had variable ability to form biofilm. We found that the highest percentage of strong biofilm producers was among the strains isolated from sputum and tracheostomy tube (66.7), nose and catheter (50%), throat (44.4%), and bronchoalveolar washings (43.8%). This indicates that production of strong biofilm by* S. aureus* isolated from respiratory tract and medical devices is a result of environmental selection that led to predominance of strong biofilm producers, because biofilm formation is important for their persistence in these difficult environmental conditions. Bridier et al. (2010) [20] reported that* S. aureus* strains from different sources produced biofilms with high biovolumes. In present study, we compared biofilm biomass and revealed that strains isolated from bronchoalveolar washings produced significantly more biofilm than strains isolated from wound and anus. Biofilm formation depends on many factors such as environment, availability of nutrients, and above all the presence of the biofilm-associated genes and their expression [21]. In our previous studies we found that the expression levels of genes encoding binding factors (elastin-, laminin-, and fibrinogen-binding protein) in weakly adhering strain of* S. aureus* were significantly lower than in strongly adhering strain of* S. aureus* [22]. The results reported by Beloin et al. (2006) [23] showed that the nature of the strains also plays role in the expression levels of genes that are involved in the synthesis of PIA. The results of our previous study revealed that in biofilm formed by weakly adhering* S.aureus* strain* icaA* expression gradually increased, while expression of* icaD* did not change over time, whereas in strongly adhering strain, the highest expression levels of these genes were detected during first hours of biofilm formation [22].

Fecal screening for* S. aureus* is of key importance in infection control and that is why in our research strains from anus of hospitalized patients constituted a large group.

Other authors showed that feces of hospitalized patients are an important source of both MRSA and MSSA strains for nosocomial transmission and a risk factor for disease development [24]. Patients with MRSA colonized diarrheal stools impact significantly on environmental contamination. In our study, 62.5% of* S. aureus* strains from feces were resistant to methicillin. Other authors showed also that hospitalized patients with stools and nose colonized are significantly more sensitive to colonize skin compared to patients with nose colonization only [25, 26]. In our research, almost all strains isolated from anus produced biofilm but the ability of biofilm forming by these strains was significantly lower comparing to strains isolated from other clinical materials. In this study we showed that the majority of MRSA and MSSA strains were moderate producers of biofilm which is in accordance with the results obtained by Smith et al. (2008) [27]. However, these authors detected no significant correlation between susceptibility to methicillin and biofilm formation, while we found that MRSA strains produced significantly more biofilm than MSSA strains. Our data were in accordance with the results obtained by Neopane et al. (2018) [21] who observed that among biofilm producers MRSA strains comprised 43.3% and no MRSA strains were identified among biofilm nonproducers. Batistão et al. (2016) [28] showed that all strains characterized as strong biofilm producers carried the SCC*mec* type III (staphylococcal cassette chromosome* mec*), while Lim et al. (2013) [29] found SCC*mec* type III as a genetic marker of strong biofilm producers.

*S. aureus* biofilm formation depends on the production of an intercellular polysaccharide adhesin named PIA which is composed of beta-1,6-linked N-acetylglucosamine residues and an anionic fraction with a lower content of non-N-acetylated D-glucosaminyl residues [30]. The products of the* ica* locus comprising* icaADBC* genes were demonstrated to be necessary for biofilm formation. The PIA mediates intercellular adherence and accumulation of multilayer biofilms [31]. In our study the* ica* operon was present in all* S. aureus* strains but strains differed in biofilm biomass. It is suggested that these biofilm producer strains also used other systems to form biofilm such as the protein A (SpA),* S. aureus *surface proteins G (SasG), and C (SasC) or the fibronectin-binding proteins (FnBPs) which were found essential for biofilm formation [32–35]. Moreover, biofilm-associated protein (Bap) and Bap-related proteins of* S. aureus* also can substitute PIA-mediated biofilm development in* ica*-independent biofilms [36]. In our research, not all genes of* ica* operon were detected in investigated strains. Similar results were obtained by Diamond-Hernandez et al. (2010) [37] and Bazari et al. (2017) [38]. In our study, statistical analysis showed that biofilm biomass of strains with* icaABCD* and* icaABD*, which formed strong biofilm, was highly significantly higher than the biofilm biomass of strains with* icaAD*. IcaB is the deacetylase responsible for deacetylation of poly-N-acetylglucosamine. Deacetylation of the polymer is essential for biofilm formation. Deletion of* icaB* leads to synthesis of complete poly-N-acetylglucosamine, less efficiently binding to the bacterial cell surface which leads to reduction in biofilm formation [39]. In this study,* icaABCD* was significantly associated with MRSA strains that had significantly higher ability of biofilm formation than MSSA strains. The prevalence of* icaABD* was similar in both MRSA and MSSA strains. Lower biofilm biomass of MSSA strains can be caused by a less frequent occurrence of* icaABCD* in this bacteria than in MRSA strains. The results obtained by other authors [40] showed that biofilm development in MSSA is* icaABCD *dependent and environmental regulation may play an important role in* ica* transcription. Biofilm development in MRSA is* ica* independent and involves a protein adhesin regulated by the accessory gene regulator (*agr*) and the staphylococcal accessory regulator (*sarA*), whereas* sarA* regulated PIA plays more important role in MSSA biofilm development [41, 42].

## 5. Conclusions

The present study revealed that MRSA and MSSA strains isolated from clinical materials from hospitalized patients produced biofilm. Biofilm biomass formed by strains from different clinical materials was different. The strains from bronchoalveolar washings produced significantly more biofilm than strains from wounds and anus, while biofilm biomass formed by strains from blood was not significantly lower than biofilm biomass of strains from bronchoalveolar washings. The ability of biofilm forming by strains from feces was significantly lower comparing to strains isolated from other clinical materials. Biofilm biomass of MRSA strains was significantly higher than biofilm biomass formed by MSSA strains. All strains had* ica* operon, and strains forming strong biofilm with* icaABCD* and* icaABD* produced highly significantly more biofilm than strains with* icaAD*. The biofilm-forming capacity of MRSA and MSSA strains indicates high ability of these strains to persist in hospital environment and increase the risk of disease development in hospitalized patients.

## Figures and Tables

**Table 1:** Oligonucleotide primers used in the study.

Primers	Sequence (5′→ 3′)	Amplicon length (bp)	References
*icaA* (F)	ACACTTGCTGGCGCAGTCAA	188	[14]
*icaA* (R)	TCTGGAACCAACATCCAACA		
*icaB* (F)	GTCTTCATTTGGAGGATTCGGC	900	[15]
*icaB* (R)	AATCACTACTGACTTCGGCTGG		
*icaC* (F)	ATGGGACGGATTCCATGAAAAAGA	1100	[15]
*icaC* (R)	TAATAAGCATTAATGTTCAATT		
*icaD *(F)	ATGGTCAAGCCCAGACAGAG	198	[14]
*icaD *(R)	AGTATTTTCAATGTTTAAAGCAA		

**Table 2:** Ability of *S. aureus* strains to produce biofilm on polystyrene.

Origin of strains (no.)	No. (%) of strains		
	Specific biofilm formation		
	Strong	Moderate	Weak
Bronchoalveolar washings (32)	14 (43.8)	15 (46.8)	3 (9.4)
Wound (25)	7 (28)	12 (48)	6 (24)
Anus (16)*∗*	2 (12.5)	9 (56.2)	4 (25)
Blood (13)	5 (38.5)	7 (53.8)	1 (7.7)
Nose (8)	4 (50)	4 (50)	-
Purulence (6)	2 (33.4)	4 (66.6)	-
Urine (5)	1(20)	4 (80)	-
Throat (9)	4 (44.4)	5 (55.6)	-
Sputum (3)	2 (66.7)	1 (33.3)	-
Tracheostomy tube (3)	2 (66.7)	1 (33.3)	-
Catheter (2)	1(50)	-	1 (50)
Other (8)	4 (50)	2 (25)	2 (25)
Total	48 (36.9)	64 (49.2)	17 (13.1)

*∗* - One strain did not form biofilm.

## Data Availability

The data used to support the findings of this study are available from the corresponding author upon request.
